# Hydrodynamic controls on connectivity of the high commercial value shrimp *Parapenaeus longirostris* (Lucas, 1846) in the Mediterranean Sea

**DOI:** 10.1038/s41598-019-53245-8

**Published:** 2019-11-15

**Authors:** Giovanni Quattrocchi, Matteo Sinerchia, Francesco Colloca, Fabio Fiorentino, Germana Garofalo, Andrea Cucco

**Affiliations:** 1 Institute for the study of Anthropic Impact and Sustainability in marine environment, National Research Council, Oristano, Italy; 2 Institute for Biological Resources and Marine Biotechnologies, National Research Council, Mazara del Vallo, Italy; 3 Integrative Marine Ecology Department, Stazione Zoologica Anton Dohrn, Naples, Italy

**Keywords:** Ecology, Ocean sciences

## Abstract

In the Strait of Sicily (SoS), a wide passage of the Mediterranean Sea, *Parapenaeus longirostris*, (Lucas, 1846; DPS hereafter) is the main target species of trawl fisheries, with an estimated annual market value of about 80 million euro. The exploitation of this resource is shared between Italian, Tunisian and Maltese bottom trawlers and its management raises social, economic and environmental interests. Recent stock assessment revealed high fishing mortalities and low size at first capture, thus promoting the adoption of a strategic plan for a sustainable management. However, the current knowledge of the geographical boundaries of the stock, supporting  the implementation of such management plan is still poor. In this respect, under different hydrodynamic regimes, particle-tracking modelling was used to explore connectivity between both, known and unexplored, spawning and nursery areas of DPS in the SoS. Ensembles scenarios derived by model outcomes displayed decadal changes in connectivity between spawning and nursery areas in the north side of the SoS, hence confirming the presence of a single stock in this area. Expanding the area of investigation, the model results showed weak connectivity between spawning ground in the north side of SoS and nurseries on the African shelf-break. This method could support the spatial management of the stock, such as the protection of the nursery and spawning areas, by providing estimates of how connectivity is influenced by hydrodynamic regimes at different temporal and spatial scales.

## Introduction

For marine species, recruitment to adult habitat is a complex process acting, with variable intensity, between the egg and the juvenile stage. It is considered as the most important natural process causing the fluctuation of marine populations and, consequently, the main forcing of the future-year age classes structure^1,2^. The success of recruitment (i.e. the amount of offspring produced that survive to join the adult population) is controlled by a number of factors, such as dispersal processes by sea currents^3^, larval and recruits behaviour, food availability and predation^4^.

In the marine environment, dispersal processes and related connectivity between spawning and nursery regions can be investigated by several methods, including analysis of spatial distribution of eggs and larvae, genetics, otolith shape and microchemistry, tagging and tracking and dispersal modelling^5^. Each of these techniques is informative about stock structure at different spatial and temporal scales^6^. Genetic analyses may provide insights on the spatial and temporal evolution of genetic composition of the stocks over several generations in the past, otolith isotopes ratios analysis give information on the averaged chemical environment over the lifespan of the fish. Tagging and tracking technique provides accurate information on the movement of fish over a temporal scale of few years and on a spatial scale of up to 100 s km^7^, however it is of limited applicability for small larvae.

To some extent, these methods have been used to estimate connectivity, but they are rarely used jointly. However, they do not measure the same thing, and the ability to reproduce dispersal of larvae may differ^8^. It would be advisable to adopt a multidisciplinary approach combining the different techniques in the context of fishery stock management^7^.

Numerical simulations, based on particle-tracking models (PTM) forced by sea currents fields, are considered an efficient approach to describe, with remarkable accuracy, the dispersal of eggs and larvae in wide areas^9^. In fact, oceanographic models can provide synoptic current fields covering seasonal and multi decadal periods, while PTM can be also designed to reproduce relevant behaviour of larvae during their pelagic phase (e.g. vertical migration).

**Figure 1:**
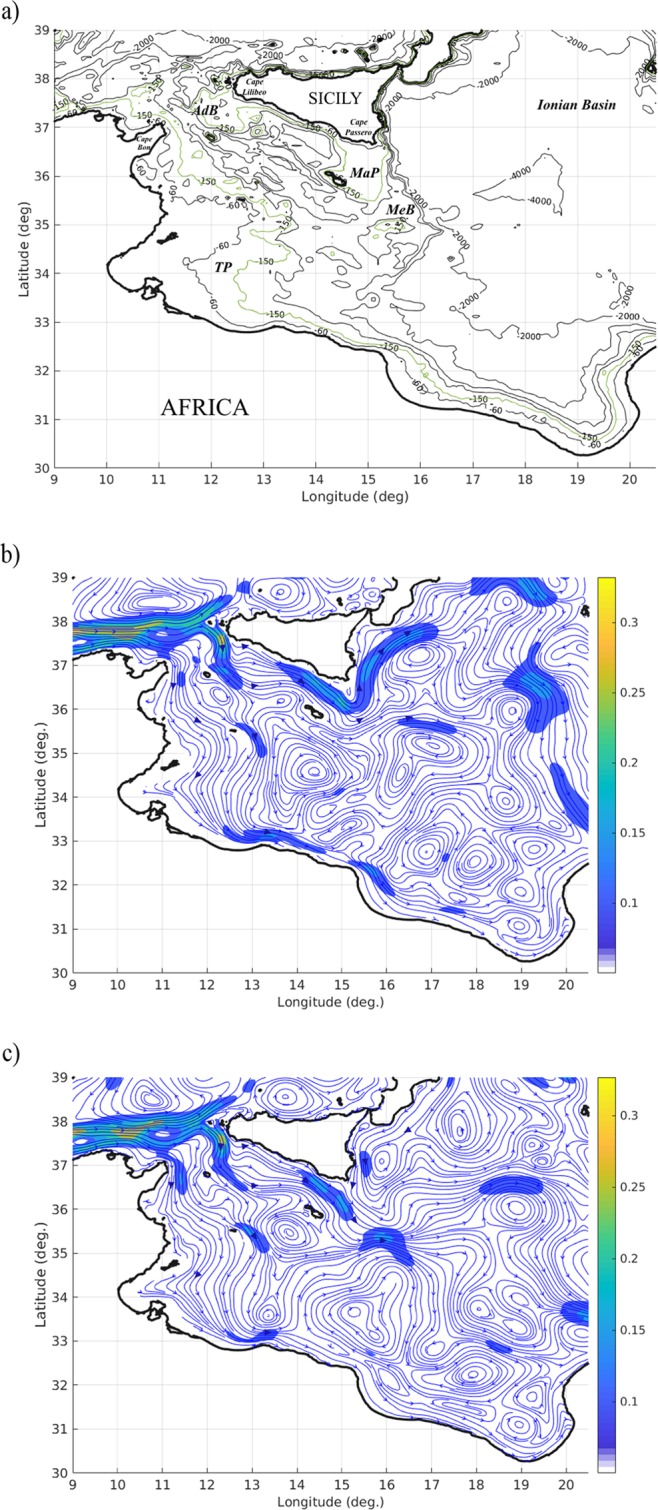
(**a**) Coastlines and bathymetry of the Mediterranean Sea, including the SoS. Light green contours (−150 m) describe the limits of the Malta Plateau (MaP), Medina Bank (MeB), Tunisian Plateau (TP) and the Adventure Bank (AdB). (**b**) Sea surface current system climatology (1994–1997) and (**c**) Sea surface current system climatology (1998–2010) of the Mediterranean Sea, derived by model hindcast. Colours palette show values of the sea current grater than 0.05 m/s.

In the Mediterranean Sea several studies were performed on the larval drift of commercially important marine species. As an example, at basin scale, the dispersal of eggs and larvae of the European anchovy (*Engraulis encrasicolus*, Linnaeus, 1758) was extensively studied in the Western Mediterranean Sea^10,11^, in the Adriatic Sea^12^ and in the south-central Mediterranean Sea^13,14^. In the Western Mediterranean Sea, at sub-basin scale, the larval dispersal of giant red shrimps (*Aristaeomorpha foliacea*, Linnaeus, 1758) was explored providing distribution and connection scenarios by means of a PTM^15^. The authors reported a strong connectivity between the western and southern sides of the Sardinia Island (Italy), while the eastern side of the sub-basin was relatively isolated. In the Strait of Sicily (SoS), the wide passage connecting the eastern and western sub-basins of the Mediterranean Sea (Fig. 1a), larval drifting between spawning and nurseries areas of red mullet (*Mullus barbatus*, Linnaeus, 1758) populations was also investigated^16^. There, planktonic stages were represented as Lagrangian drifters released in the known spawning areas, providing both, an estimate of the possible linkages between the Sicilian–Maltese and the African sides of the SoS and a preliminary overview of the relationships between population subunits in the area.

Deep-water rose shrimp (*Parapenaeus longirostris*, Lucas, 1846; DPS hereafter) is the main target species of trawl fisheries with landings representing about the 50% of the whole species catches in the Mediterranean and recent Sicilian production varying between 5,000 and 10,000 tonnes annually, with an estimated annual market value up to 80 million euro^17^. FAO stock assessments^18^ evaluated that it is affected by high fishing mortalities and low size at first capture. Despite its economical importance, knowledge about the stock structure of the species in the SoS and in the adjacent areas is still poor^19^. Such knowledge is extremely important for the future management of the stock also considering that DPS has been proved to be extremely sensible to the on-going warming trend of Mediterranean waters with a positive response of populations to the increasing of temperature^20^. Already in 2011^21^ SoS was defined a priority area for conservation and an Ecologically or Biologically Significant Area (EBSA) in 2014^22^. Indeed, the SoS (Fig. 1a) is an important biodiversity hotspot of the Mediterranean Sea^23^, where complex geomorphology and oceanography frame distinctive habitat. There, water of Atlantic origin dominates the sub-surface ocean circulation with the Atlantic Ionian Stream (AIS). In the northern part of the SoS, this current is flowing from west to east and, interacting with continental margins and banks, generates gyres and upwelling affecting bottom substrates and fishes population dynamics^24^.

To sustainably manage the DPS stocks, in May 2016^25^, a management plan, not yet fully enforced, has been developed to protect important nursery areas in the SoS. Since an exhaustive definition of the stock structure and its geographical boundaries has not yet been realized, the authors aim at filling the gap in the knowledge of the DPS spatial stock structure of the SoS by investigating the larval aggregation close to settlement and its variability, as forced by main hydrodynamic constraints, into both known and unexplored nurseries of the SoS.

Specifically, this study pursues two main objectives: first, to verify how low-frequency (e.g. decadal) ocean variability in Mediterranean Sea affects DPS recruitment into known nurseries of the northern part of the SoS, second, to verify whether recruitment of DPS into known and unexplored habitat of the whole SoS domain may have originated from spawning areas in the northern part of the SoS.

The methodological approach involves the adoption of a long-term dataset (2003–2010) of scientific fishery and oceanographic hindcast data (1994–2010). The former previously used to identify DPS spawning and nursery areas^26,27^ in the SoS and the latter to describe the dynamics of the main sea currents routes in the same region. Beside, geographical information on bathymetry and substrate were used to identify further potential areas for DPS recruitment^28^ while a PTM, forced by oceanographic hindcast data, was used to reproduce multi-year larval dispersal of DPS in the SoS.

Considering that the northern part of the SoS is a known DPS spawning area^27,28^, this was selected as the release area for numerical particles in two PTM experiments that were set up. The first experiment reproduced the larval aggregation within the boundaries of known nursery areas located in the northern part of the SoS. Model results were compared to survey data and hypotheses of connectivity were drawn. The second experiment, including all DPS suitable habitats in the SoS, derived by bathymetric and substrate preferences, reproduced the larval aggregation into known and unexplored habitat, investigating whether and how, at long distances, the observed Sicilian-Maltese spawning areas could act as a source for recruitment areas of the whole SoS.

The PTM simulations have been carried out for the period in between 1994 and 2010. We considered that larvae are capable of settling to a nursery ground at the end of the larval phase, which was reported to occur between few days and two months from the spawning, as stated for DPS^29^ and for diverse paeneids shrimps species^30,31^. For each year two simulation were performed, and then combined each one considering two potential time windows for particles recruitment: 10–30 and 40–60 days after particle release. Time window 10–30 represents a summer spawning with consequent fast larvae development while time window 40–60 represents a winter spawning with consequent slow larvae development.

According to the occurrence of the twain known states of the Northern Ionian Reversal phenomenon (NIR)^32^ that affects, with a decadal periodicity, the SoS sea current system, PTM simulations were then combined along the temporal dimension to produce ensembles scenarios: ENS 01 for the simulation period 1994–1997, ENS 02 for the simulation period 1998–2010, ENS 03 for the simulated year 1995 and ENS 04 for the simulated year 2004.

In fact, decadal changes in the sub-surface sea current system of the south-central Mediterranean Sea were recently described as the NIR^32^ and can be here explained by Fig. 1b,c, where model derived climatologies are showed. The NIR is a current reversal that takes place in the northern Ionian Sea and affect the eastern part of the SoS current system. During a first state, characterizing the years in between 1994 and 1997 (Fig. 1b), an intense Atlantic Ionian Stream outflows into the northern part of the Ionian Sea and it gives rise to an overall anticyclonic circulation. During a second state, occuring between 1998 and 2010, (see Fig. 1c) the circulation in the northern Ionian Sea is cyclonic and a weaker AIS cut across the Ionian Sea at the latitude of around 36°N^32,33^. In the following we will refer to NIR-1 and NIR-2 when considering the two states characterizing the NIR.

The PTM results were analysed through two main indices, a larval aggregation index (LA hereafter) and particles fluxes (PFs hereafter). LA is an index of recruiting particles and it is computed as the fraction of the larvae that recruit over the total amount of larvae born annually. Its values were split into five classes, very low (0.05–0.20), low (0.21–0.40), intermediate (0.41–0.60), high (0.61–0.80) and very high (0.81–1). PFs were computed to describe the origin of recruited particles and to identify the amount of particles released from a specific area reaching to another area during the simulated period (see methodological section for indices’ definition and computation).

Within the limit of the numerical model approach, given by a generalization and simplification of the physical model, LA and PFs provided information on connectivity between known spawning and potential nursery areas of DPS in the SoS, useful for the identification of the species’ stock structures. A decadal variability of connectivity was found and results were discussed in relation to decadal features of SoS hydrodynamics.

## Results

### Exp. 1 - Results for known DPS nursery areas

In the first experiment the PTM run for all years between 1994 and 2010. The results were combined into ensembles, ENS 01 (1994–1997) and ENS 02 (1998–2010), which periods coincide with the occurrence of NIR-1 and NIR-2^32^, respectively.

As detailed in the methodological section, numerical particles were released into known spawning areas of the northern part of the SoS and advected by the PTM until they reach recruitment areas located in the northern region. Spawning and nursery areas, inferred by the observational dataset, were grouped into three macro regions called WEST, EAST and S- EAST in order to simplify the representation of model outcomes and discussion.

**Figure 2:**
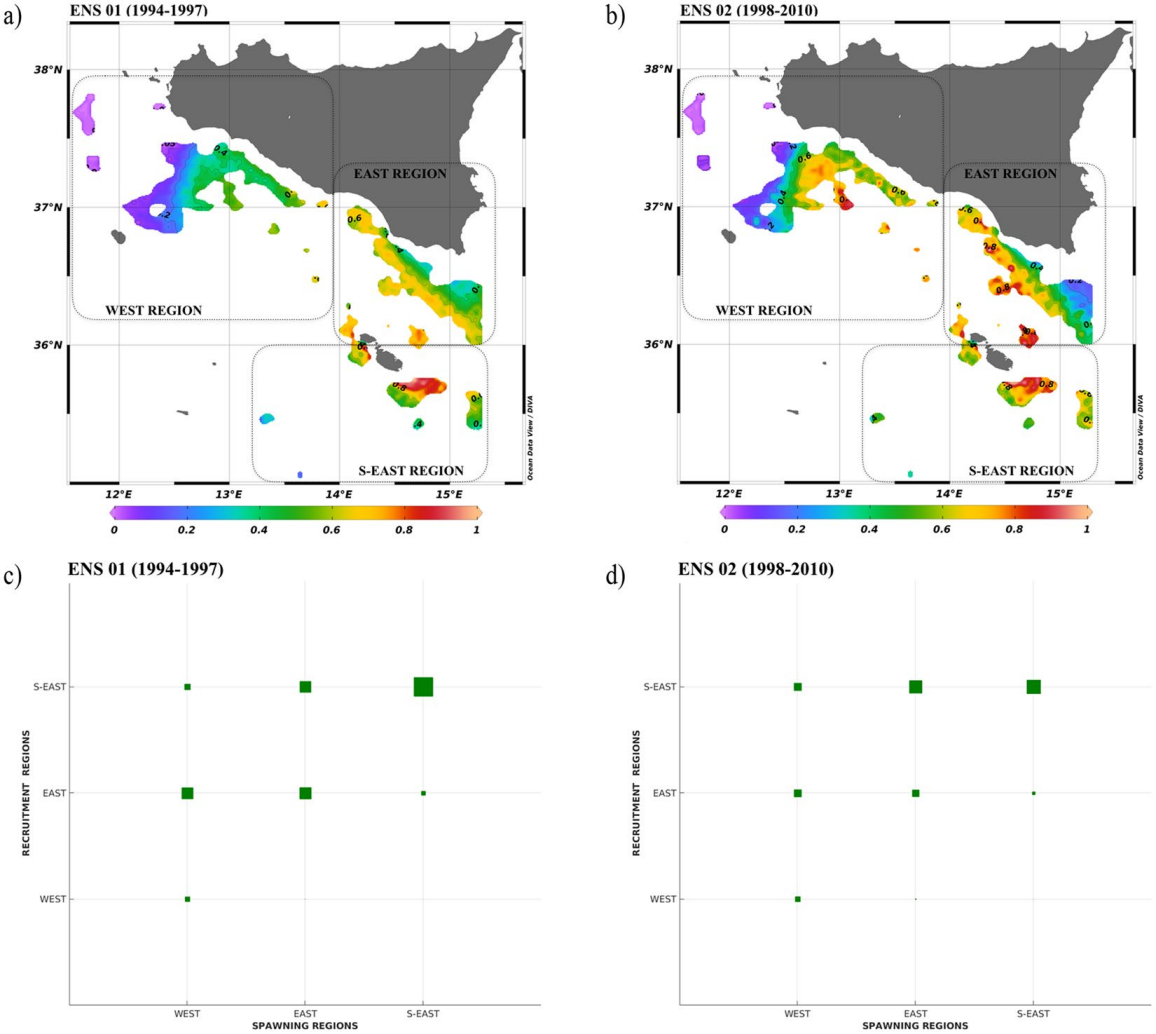
(**a**) Larval Aggregation index map (LA) as generated by the ensemble *ENS 01* (1994–1997). (**b**) LA map as generated by the ensemble *ENS 02* (1998–2010). (**c,d**) Graphical representation of the PFs from spawning to recruitment regions. Abscissa/Ordinate axes indicate the name of the spawning/recruitment regions in Fig. 1. The bigger is the green square the higher is the PF. Left and right panels refer to the ensemble *ENS 01* (1994–1997) and ensemble *ENS 02* (1998–2010), respectively.

The PTM results are displayed in Fig. 2a,b through the spatial distribution of the LA index, obtained for ensembles ENS 01 and ENS 02.

ENS 01 accounts for the effects induced by NIR-1 oceanographic state (i.e. intense AIS flowing towards the northern part of the Ionian basin). In this case LA was low and intermediate in the WEST region and it was intermediate and high in the EAST region. S-EAST region showed maxima of LA ranging between high and very high. Thus, ENS 01, due to the effects of the sea current regime in NIR-1, displayed a rapid eastward dispersion of released particles, shifting potential settlement and aggregation chances into the known easternmost DPS nurseries of the SoS and reducing the recruitment process on the WEST region.

ENS 02 accounts for the effects induced by different hydrodynamic conditions due to the switch to NIR-2 (i.e. slightly less energetic AIS that flows into the Ionian basin at lower latitudes). Intermediate LA values were found in all recruitment regions with exception of the westernmost portion of the WEST region and the north-easternmost portion of the EAST region, displaying low LA. EAST and S-EAST recruitment regions were characterized by high and very high LA, specifically around the Maltese archipelago and along a strip close to the Sicily coasts. Under ENS 02 significant larvae aggregation occurred over the entire set of the known DPS nurseries of the SoS with noticeable hotspots in correspondence of the Adventure Bank, in the WEST region, and the Maltese Plateau, in the EAST region.

Considering PFs during both states of NIR, computed from ENS 01 and ENS 02, Fig. 2c,d display that the recruitment region WEST is independent from spawning regions EAST and S-EAST and that recruitment in the EAST and S-EAST regions strongly depend on local spawning. PFs from the spawning region WEST always affect recruitment in the recruitment regions EAST and S-EAST.

The maximum value of PFs was found between S-EAST spawning and S-EAST recruitment regions. Long-distance connections were identified between WEST region, where the Adventure Bank is located, and EAST and S-EAST regions, corresponding to the Maltese Plateau. This suggests that recruitment in these regions is not independent but, instead, also controlled by variable hydrodynamic features.

In ENS 02 (Fig. 2d), with the exception of the WEST region, recruits of local origin appeared less pronounced. The PFs indicate that particles move with similar intensity from spawning region WEST towards recruitment regions EAST and S-EAST. Spawning region EAST was well linked to recruitment region S-EAST. ENS 02 also showed the maximum value of PF between S-EAST spawning and S-EAST recruitment regions.

**Figure 3:**
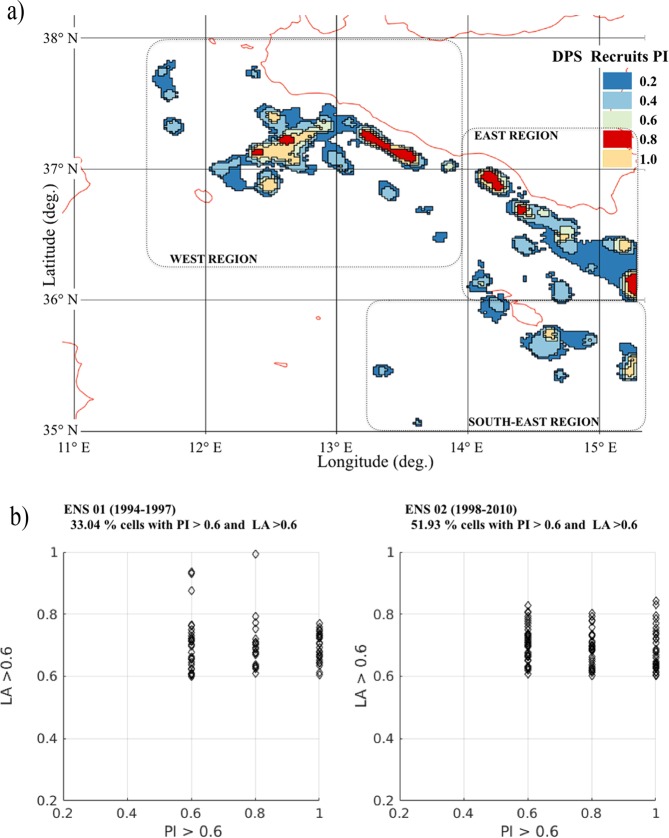
(**a**) Persistence Index (PI) map computed for DPS recruitment areas reporting five classes of persistence (adapted from^30,38^). (**b**) Geographic elements of the recruitment areas that are showing PI and LA greater than 0.6. Right and left panels refer to the ensembles *ENS 01*(1994–1997) and *ENS 02* (*1998–2010*), respectively.

A qualitative comparison of LA with trawl survey abundances can be carried out through a map of Persistence Index (PI) of DPS recruitment^27^ in the northern part of the SoS (Fig. 3a). Results, encourages the adoption of this set up for this and further experiment Exp. 2. PI is derived by a 2003–2010 trawl survey dataset and, ranging between 0 and 1, it indicates the total absence of DPS recruits density hot spots or the maximum time persistence of DPS recruits density hot spots throughout the years^28,34^.

PI, derived by recruit abundance indices (Fig. 3a), and LA, derived by model simulations (Fig. 2a,b), revealed a spatial correspondence between recruitment hotspots, defined as those areas with LA or PI greater than 0.6. This was particularly evident in the WEST region, in correspondence of the Adventure Bank and the Sicilian shelf-break, in the northern part of the EAST region and in the S-EAST region.

Figure 3b, left and right panels, provides a quantitative comparison between PI and LA recruitment hotspots obtained for ENS 01 and ENS 02, respectively. The graphs report the number of model grid cells in which LA values and PI values are both greater than 0.6 (i.e. hotspots). For ENS 01 (1994–1997) this comparison revealed a 33.04% of overlapping recruitment hotspots cells whereas, for the ENS 02 (1998–2010), in which simulated years include the scientific trawl surveys period (2003–2010), model results significantly improved with a 51.93% of overlapping recruitment hotspots cells.

### Exp. 2 - Results for known and unexplored DPS nursery areas

In the second experiment the PTM run for the years 1995 and 2004. These years were considered as representative of the two distinct states of the NIR (NIR-1 and NIR-2) affecting, with decadal occurrence, the AIS configuration. The model setup assumed known spawning areas in the northern sector of SoS and both known and unexplored nursery areas, hence including all potential DPS nurseries in the south-central Mediterranean including those of the southern sector of SoS.

**Figure 4:**
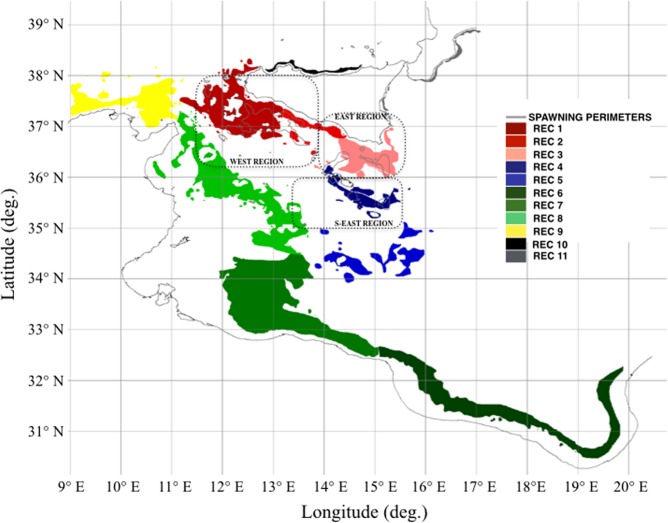
Geographical distribution of the potential recruitment areas defined for the SoS and adopted in the setup of the second experiment. Colours identify the geographical location of the recruitment regions. The light grey polygons define the boundaries of spawning areas from which numerical particles are daily released, the same adopted for the first experiment.

Figure 4 shows spawning areas (grey polygons) from which numerical particles are released daily. They are located in the northern part of the SoS and they are the same of the first experiment. It also shows the geographical extent of potentially suitable recruitment areas (i.e. nurseries), defined by adding to the already known nursery areas those satisfying the habitat suitability criteria for DPS settlement (mud substrates and depth in the range 100–300 m)^35^. In order to clearly discuss model outcomes, suitable recruitment areas were grouped into eleven recruitment macro regions (REC): REC 1, REC 2, and REC 3, located in the northern part of the SoS from west to east, REC 4 and REC 5, located nearby the south part of the Malta archipelago and the Medina Bank, respectively. Regions named REC 6, REC 7, REC 8 and REC 9 identify four recruitment regions along the North African shelf-break from east to west. Regions REC 10 and REC 11 (north and east side of Sicily island) satisfy the habitat suitability criteria but they are not located within the SoS domain.

**Figure 5:**
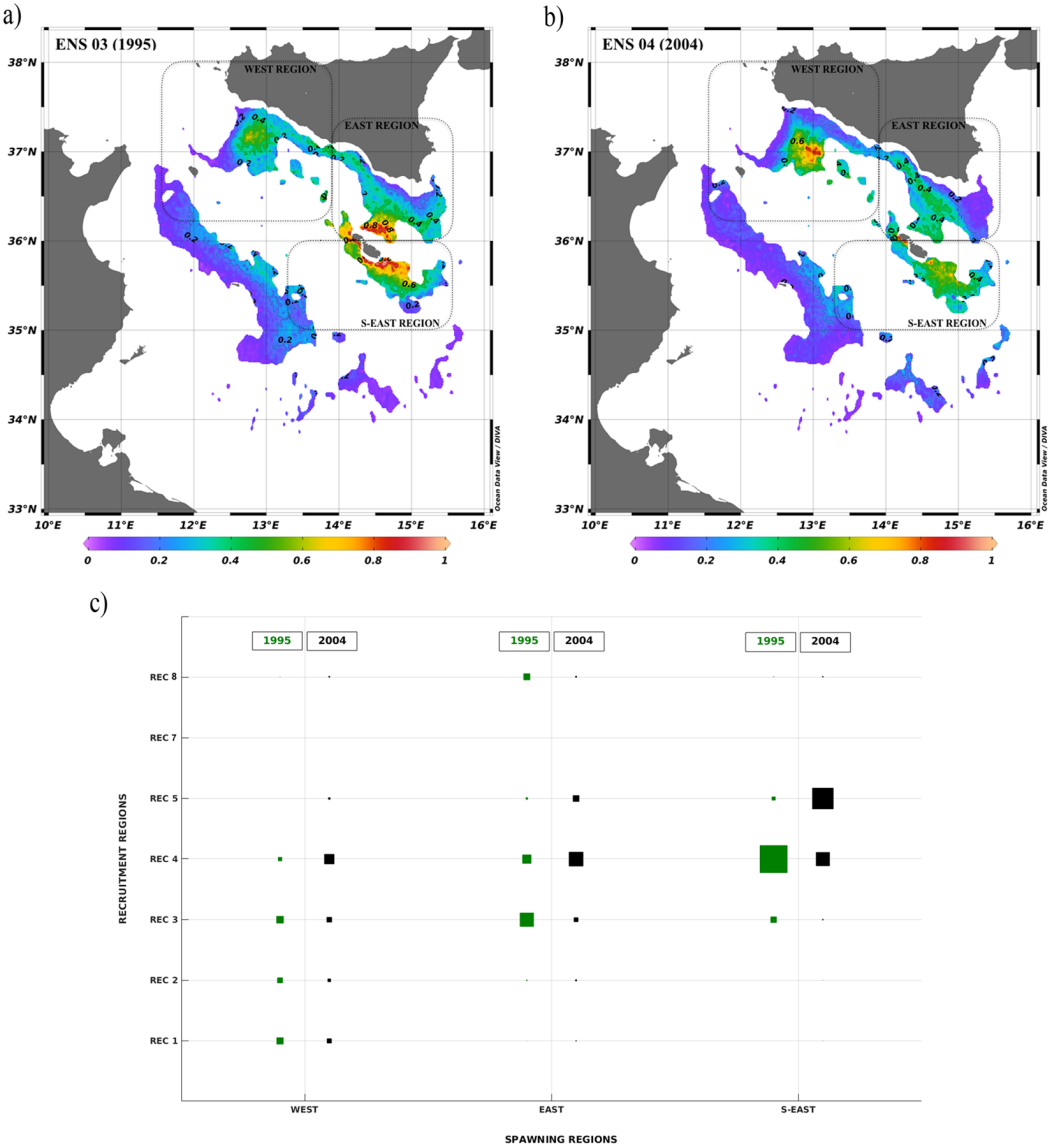
(**a**) LA map as generated by the ensemble *ENS 03* (1995). (**b**) LA map as generated by the ensemble *ENS 04* (2010). (**c**) Graphical representation of the PFs from spawning to recruitment regions. Abscisse indicate the name of the spawning regions (Fig. 1), ordinate indicate the name of recruitment regions (Fig. 4). The bigger is the square the higher is the PF. Green squares refer to the ensemble *ENS 03* (1995) and black squares to ensemble *ENS 04* (2010).

Results from the PTM simulations were combined into ensembles of LA: ENS 03 for 1995 and ENS 04 for 2004. Figure 5a,b display, for ENS 03 and ENS 04, the geographical distribution of LA in the above-defined recruitment regions. These maps display LA values greater than 0.05, excluding recruitment regions from REC 8 to REC 11 characterised by minor values of LA.

The results from ENS 03 scenario, representing the effects of NIR-1 on the SoS sea current system, displayed low to intermediate LA, in the western side of the SoS (REC 1 and REC 2), whereas high to very high LA was found in correspondence of recruitment regions REC 3 and REC 4 at the East. Maxima of LA, over 0.8, were found around the Maltese archipelago and intermediate to high LA on the east edge of the Maltese Plateau, off Cape Passero. Low LA was found along the Tunisian shelf break, spanning between longitudes of 12°E and 13.5°E and corresponding to recruitment region REC 7. REC 5 displayed very low LA (0.05–0.2).

In ENS 04 scenario, representing the effects of NIR-2, the LA map displayed hot spots with high and very high LA on the western side of the SoS, in correspondence of the Adventure Bank and its surroundings (REC 1). Intermediate LA was found in REC 2 and REC 3 within the Maltese Plateau. LA in REC 4 ranged between intermediate to very high, with hotspots in correspondence of the southern coasts of the Maltese Plateau. Very low and low LA was found in correspondence of REC 5 and REC 6. Low LA, with maxima of 0.4 was found around 13.5° E and 35.5° N in correspondence of the Tunisian shelf-break (REC 7). The main differences between ENS 03 and ENS 04 scenarios occurred around the Adventure Bank and the Maltese Plateau. In both simulations, the easternmost part of the Adventure Bank displayed very low LA.

Both ensembles revealed low and intermediate LA in the neighbouring of Medina Bank and along the Tunisian shelf-break.

PFs, computed for ENS 03 and ENS 04 are reported in Fig. 5c. The graph shows the linkages between spawning macro regions (WEST, EAST and S-EAST) and 7 of the 11 recruitment macro regions (from REC1 to REC7). The dimension of the squares represents the intensity of the PFs and their colours indicate the simulated periods, green for 1995 and black for 2004.

Considering both computed periods, the spawning region WEST was found to be connected to the closer recruitment regions, REC 1 and REC 2, and faraway regions, REC 3 and REC 4, in the ratio of 13% and 25%, for the 1995, and 13% and 46%, for 2004, of the maximum computed PF per ensemble. In 2004 the connections from this spawning region included, at lower intensity, also regions REC 5 (8%) and REC 7 (5%), located off the Tunisian coast.

In 1995, spawning region EAST was weakly connected to regions REC 1 and REC 2. High connectivity was instead found in correspondence of regions REC 3 (49%) and REC 4 (31%), indicating that most of the recruited larvae were spawned within short distance. PFs were found with region REC 7 (22%), off the Tunisian coast. Similar results were found in 2004, with the most intense connection (67%) in correspondence of REC 4.

Spawning region S-EAST displayed a local retention of numerical particles that indeed converged in regions REC 4, in 1995, and REC 4 and REC 5, in 2004. Low PFs to REC 7 were identified during both years (1–4%).

The main changes in advection between the two states of the NIR are reflected in the antithetic PFs displayed during 1995 and 2004, for all linkages between all spawning regions and recruitment regions REC 3, REC 4 and REC 5. Low PFs were found between all spawning regions and recruitment REC 8, except in 1995 between EAST spawning regions and REC 8. Regions REC 7 showed negligible values and REC 6, 9, 10 and 11 did not display evidence of LA.

## Discussion

For stock assessment and fishery management purposes DPS in the Strait of Sicily (SoS) is considered a stock whose life cycle occurs between coastal and offshore waters and whose catches are shared between Italian, Tunisian and Maltese bottom trawlers^17^. For these reasons information on species spatial distribution and connectivity acquires a high importance to improve effectiveness of management measures^36^.

This study presents the application of a particle-tracking model (PTM), characterized by long-term simulations, where hydrodynamic constraints provide connections between observed spawning areas, in the northern part of the SoS, and known and unexplored nursery areas of DPS in the whole SoS domain. Such knowledge of connectivity, and how it varies in response to changes in environmental conditions, can provide an useful hint for the future spatial management of the stock centred on spatial based measures, such as the protection of the nursery and spawning areas. However it is worth noting that actual settlement of fish or shellfish recruits to nursery grounds is considered to be not directly proportional to the number of larvae transported over suitable nursery areas, as this depends on a number of factors linked to larvae survival (e.g. behaviour, competency, predation, etc.)^7^. After all if larvae are transported away from suitable nursery areas, they certainly will not be able to survive.

In particular, model results revealed the role of decadal variability of the south-central Mediterranean Sea circulation, favoured by the Northern Ionian Reversal phenomenon (NIR)^32^.

Indeed, the PTM simulations in experiment EXP 1, investigating connectivity between known spawning and nursery areas both located in the northern sector of the SoS, shown a strong variation of the spatial pattern of recruitment due to the effects of the NIR occurrence.

During the first state of the NIR, before 1998, long-distance connections were identified between WEST region, where is located the Adventure Bank, and EAST and S-EAST regions, corresponding to the Maltese Plateau. Conversely, during the second state of the NIR, after 1998, local higher retention of particles was found in the WEST region and connections between the WEST and the EAST regions were reduced in favour of the S-EAST region, which may be due to the southward shifting of the AIS jet.

In both cases, recruitment in WEST region seems to be independent from EAST and S-EAST spawning regions and that recruitment in the EAST and S-EAST regions depend on local spawning. Spawning in the WEST region always affect recruitment in the EAST and S-EAST recruitment regions.

Results obtained on the northern side of the area therefore suggested that contiguous spawning and nursery areas on Adventure Bank and Maltese Plateau are connected through larval drifting by the Atlantic Ionian Stream that is flowing in surface waters^28,37^. In 2013 a first study^19^ on the genetic structure of DPS in the central and eastern Mediterranean Sea identified some minor differences between samples from the western and eastern side of the SoS, and a general mixing in the DPS inhabiting the Northern part of the SoS that is still considered as a single stock^38^.

PTM simulations in experiment EXP 2 were also set up to verify whether the known DPS spawning areas in the northern part of the SoS can provide recruits for unexplored potential habitat in the entire domain of the SoS.

Recruitment into nurseries of the northern part of the SoS was affected by decadal changes due to the NIR occurrence. Connectivity with the southern part of the SoS was modest and mainly unaffected by the consequences of the NIR. In fact, evident changes in the climatological current fields were not found along the African shelf-break, where numerical particles are potentially advected in occasion of the AIS meandering.

For species like DPS, that experience a 1–2 months planktonic larval phase, hydrodynamics are expected to largely determine their spatial distribution^39,40^. Larvae may hence cover long distances being transported over hundred kilometres before setlement^41,42^ especially where intense current flow drives floating particles. Sensitivity experiments, considering separate ensembles for fast (10 to 30 days) and slow (40 to 60 days) larvae development scenarios, showed that pelagic larval duration modulates larval aggregation in far-off recruitment regions (see supplementary material).

In actual fact, there is only one study reporting the duration of DPS larval period to last for about two months but laboratory studies on other shrimp species swimming response to current flow^43^ show that early stages were not able to maintain position at velocities as low as 10 cms^−1^. In presence of some information on larval development, competency and settlement cues it would be possible to include this knowledge in individual based model to allow for a mechanistic response of settlement to changing environmental conditions. However, this information for DPS is not available and settlement processes were not included in the present study.

A recent study on eco-regionalization^44^, derived by connectivity analyses based on PTM simulations, describe the Mediterranean Sea current dynamics as divided into 22 zones. The SoS is divided into two main zones, North and South. The boundary between them is mostly parallel to the AIS jet that represents a semi-permanent barrier for current advection^45^. According to these authors, the boundary is only partially stable due to circulation variability, promoting north-south flow exchanges and consequent of larvae whose origin is found in spawning regions of the northern part of the SoS. AIS jet evolution in the SoS is also characterized by seasonal variability^46^ correlated to variability of the Mistral and Sirocco wind regimes in the area^47^.

Considering this second PTM experiment, although weak connectivity between the spawning areas in the northern sector of SoS and the nurseries off the Tunisian coast was found the recruitment was prominent in the northern sector. Overall a high connectivity from the spawning areas in the west side to the nurseries in the eastern ones was found, being stronger when the circulation in the Ionian Sea is anticyclonic.

These results are supported by genetic data showing minor differences for individuals from the West and East sectors of the SoS, thus confirming our connectivity results, and the presence of a gradual discrepancy along a west-east axis coherent with an ‘Isolation By Distance’ model, within the Mediterranean Sea^19^. It would be however advisable to extend the genetic analysis to African region to have a refined knowledge on the genetic pattern of DPS in the whole SoS domain, which would be clearly relevant for the management of the stock since its exploitation is shared between EU (Italy, Malta) and north African countries (Tunisia, Libya and Egypt).

Further knowledge on nurseries and spawning grounds along the African coasts by genetic analysis could support these numerical model results and the considerations that are presented here. Nevertheless, our results, predicting exchanges of larvae spawned in the Adventure Bank with nurseries off the Tunisian coast and in the south part of the Maltese Plateau, where local retention of larvae also occurs, suggest that the stock structure of DPS in the SoS could be considered as a population formed by different subunits whose connection is modulated by variability in oceanographic processes. This kind of spatial structure was already proposed for *Mullus barbatus* populations in the SoS^16^.

Wider application of spatially explicit models in future stock assessments will require clear identification of stock components, evaluating movement rates and determining the degree of reproductive isolation^48^. The knowledge on the geographical distribution of nurseries and the connectivity to spawning areas is clearly an important issue for setting the spatial scales over which fisheries should be managed and marine protected area (MPA) networks designed and implemented^49,50^. In particular, the PTM experiments on DPS revealed the key role of the WEST spawning grounds in fuelling also nurseries in the EAST and SOUTH sectors of the SoS, a new knowledge that should be considered in any future spatial management planning aimed at enhancing the productivity of the stock on the wider spatial scale of the SoS region.

Previous studies on the DPS nurseries and spawning areas distribution in the SoS have shown their high spatio-temporal persistence in relation to the main pattern of water circulation and retention structures supporting the Bakun paradigm^51^ for stock productivity that links favourable spawning habitats to the combined effect of three main oceanographic mechanisms (1) enrichment (e.g. upwelling, water mixing), (2) concentration (convergence, frontal formation, water column stability) and (3) retention within (or drift toward) appropriate habitats^27,28^. Such temporal persistence of nurseries ad spawning grounds is a prerequisite for their inclusion in a conservation network and, when combined with quantitative data on spatial connectivity, offer new opportunities to prioritize the management of DPS fisheries in the marine space^26,34^.

Furthermore, results from the PTM experiments showed that connectivity in the SoS is influenced on a decadal time scale by hydrodynamic regime shifts, which should be accounted for a dynamic management of the resources^48,52^.

In conclusion, the use of PTM provides a valuable tool for the estimation at different temporal and spatial scales of how connectivity may vary with changes in hydrodynamic conditions. With the integration of more detailed information on the biology of larval stages of DPS (e.g. temperature-dependent pelagic larval duration) this methodology could assist in the spatial management of fishery, as well as conservation objectives, by assessing the relative contribution of different spawning areas to the overall productivity of the stock.

## Materials and Methods

The adopted approach involves multiple tools and datasets:

(i) geographical information on bathymetry and substrate, were derived by EMODnet platform (www.emodnet.eu), and was used to describe complex bathymetry and identify preferential areas for DPS recruitment, typically characterized by muddy bottoms at depths between 100 and 300 meters^28^.
(ii) A long-term dataset (2003–2010), derived by bottom trawl surveys MEDITS and GRUND^53,54^, carried out in late spring (May-June), and used to provide the geographical distribution of spawning and nursery areas^26,27,55^.
(iii) A long-term oceanographic model hindcast data to define the main oceanographic configurations of the SoS.
(iv) A particle-tracking model (PTM) forced by such oceanographic hindcast data, used to reproduce multi-year larval dispersal of DPS. This model treats eggs as numerical particles subject to advection, diffusion and turbulent ocean dynamics.

### Site description

The Strait of Sicily (SoS) is a wide passage connecting the eastern and western side of the Mediterranean Sea (Fig. 1a). The geographical extension of the SoS includes complex coastal geometries and bathymetry exhibiting wide banks, plateaus (ranging between depths of 80–250 m) and an abyssal plain (with depths in between 1000 and 2000 m). On the eastern side, a submarine plateau extends from Cape Passero (Sicily island) to the Maltese archipelago (Maltese Plateau) and the Medina Bank near 35.0°N and 15.5°E, whereas on the western and southern sides the Adventure Bank and the wide North African shelf (Tunisian Plateau) characterize the SoS bottom topography.

The general sub-surface circulation of the SoS is mainly driven by the combined effect of wind stress and buoyancy fluxes that induce seasonal and decadal variability in the observed mesoscale structures^24^. A cool and low saline concentration water mass of Atlantic origin flows in the SoS with two main streams, the Atlantic Ionic Stream (AIS), meandering along the northern part of the SoS, and the Atlantic Tunisian Current (ATC), flowing along the Tunisian shelf-break. The hydrological features of these currents are progressively modified becoming warmer and saltier and, interacting with continental shelves and the margins of the main banks, they generate geostrophic gyres and upwelling affecting bottom substrate characteristics, habitats and fishes population dynamics^33^.

In Fig. 1b, 1994–1997 and 1c, 1998–2010 model derived climatologies of the sub-surface sea current system revealed decadal changes due to the recently identified Northern Ionian Reversal phenomenon (NIR)^32^. It is a current reversal that takes place in the northern Ionian Sea. In the first period (Fig. 1b), an intense AIS outflow into the northern part of the Ionian Sea gives rise to an overall anticyclonic circulation. In the second period (Fig. 1c) the circulation in the northern Ionian Sea is cyclonic and a weaker AIS cut across the Ionian Sea at the latitude of around 36°N^32,33^. During both periods quite stable meandering and recirculation systems are generated around the Adventure Bank, south of Lampedusa Island and on the West and Southwest of Malta Island, respectively. The ATC does not display significant variations.

The deep-water rose shrimp (DPS) is the main target species of trawl fisheries in the SoS and Mediterranean Sea in general. Long-term surveys provided the actual knowledge of DPS spawning and nursery areas in the northern sector of SoS^26–28,55^. Bottom substrates of these areas are characterized by coastal terrigenous mud, open-sea detrital bottoms, compacted muds and soft muds with fluid surface film; biocenosis/facies types mainly inhabited by DPS^35^.

DPS is a short-lived species characterized by high growth and mortality rates^56^, which reproduces throughout the year^57,58^. Individuals are mainly found in sandy and muddy bottoms between 100 and 300 m, although the species has a bathymetric distribution range of 20–750 m^58^. In the Mediterranean Sea, both sexes of DPS reach maturity in the first year of life^57^. After being spawned, the eggs and planktonic larval phases develop in surface waters^59^. The postlarva, similar to adults, reaches the sandy-muddy bottoms on the continental shelf and begins the bentho-pelagic cycle^28^. There is little information on the duration of the larval phase with an author reporting general information that DPS larvae can settle to the nursery grounds within two months after release^54^.

### Numerical model

Within the variety of available tools (e.g.^60^), a multipurpose PTM for larval transport named LTRANS^61^ was chosen to track, in off-line mode, numerical particles advected by stored outcomes of oceanographic models.

Hard coding of the PTM scripts allowed the adoption of a long-term set of accurate and wide-area physical data that were provided by the *reanalysis components* of the Mediterranean Forecasting System (MFS; http://marine.copernicus.eu/).

MFS consists of a hydrodynamic primitive equation model in spherical coordinates, it is implemented in the Mediterranean Sea at 1/16° (about 7 km) horizontal resolution and 72 unevenly spaced vertical z-levels^62^. The model includes a variational data assimilation scheme for historical dataset and operational observations network. The MFS domain extends into the Atlantic, where is nested with ocean climatology, and surface fluxes are derived by the 6-h, 0.75° horizontal-resolution ERA Interim reanalysis fields of the European Centre for Medium-Range Weather Forecasts.

LTRANS is based on a 4^th^ order Runge-Kutta scheme for particle advection and a random displacement model for vertical turbulent particle motion. The advection scheme solves for the current velocities at the particle location incorporating velocities at previous and future times to provide a robust estimate of particle route even in water bodies with complex fronts or eddy. The zonal and meridional current velocities provided by the MFS model and used as inputs for the Runge-Kutta scheme are multiplied by the duration of the internal time step (*dt*) in order to calculate the displacement of the particle in each component direction. Displacements are then added to the original location of the particle (*x*_*n*_, *y*_*n*_) in order to calculate the new location of the particle.

1 $$\begin{array}{c}{x}_{n+1}={x}_{n}+udt\\ {y}_{n+1}={y}_{n}+vdt\end{array}$$

In order to increase the realism of the model simulations, an ad hoc set up is adopted to reproduce the DPS pelagic larval duration and to determine whether a particle is inside or outside suitable habitat previously defined by appropriate polygons. If the particles reach a specified age for settlement the model determines whether they are inside or outside the habitat polygons; if the motion causes them to exceed the domain boundaries multiple times the particles are not anymore advected.

### Design of experiments

The PTM application included the current knowledge of DPS density hotspots of mature specimens and recruits in the SoS^26–28,55^ that was used to define the geographical distribution of spawning and nursery areas, where numerical particles, representing the DPS eggs, were released and where they could potentially settle. Eggs are then advected by the PTM until recruitment, occurring when they arrive within the boundaries of a nursery area at a suitable age for settlement. The particles were randomly released along the water column and eggs and larvae are assumed to be passively floating in surface waters (depth range 0.0–34.16 m) until settlement^63^.

Since mature females of this species have been recorded all year in the northern part of the SoS^63^, with peaks from November to February and in April and a drop in June and July^56^, the model was set up to provide continuous spawning (i.e. numerical particles release) throughout the year. The model also considers that larvae are capable of settling to a nursery ground at the end of the larval phase, which was reported to occur between few days and two months, as stated for diverse paeneids shrimps species^30,31^. Therefore, it assumes that larvae reach the minimum length for settling to a nursery ground in two time windows: 10–30 and 40–60 days after spawning. Before, they are considered too small to settle and after they are removed from the simulation.

**Figure 6:**
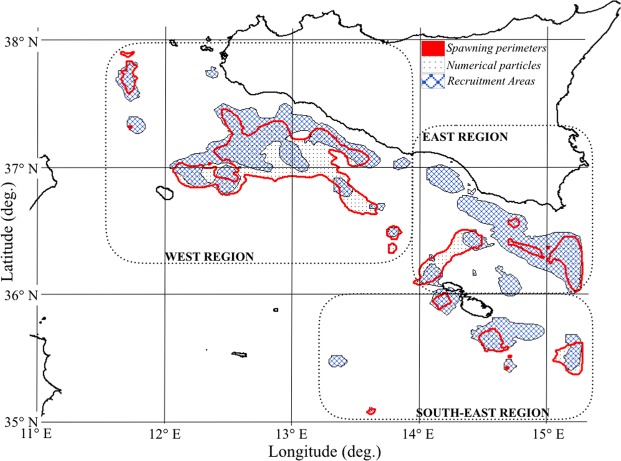
Northern part of the Strait of Sicily (SoS). Known spawning areas (red polygons) with the location of the released numerical particles (black dots). Known recruitment areas (blue crossed lines). Macro regions (dotted lines) grouping multiple areas of spawning and recruitment.

The PTM uses external time step corresponding to the daily frequency of the released physics products (i.e. zonal and meridional current velocities) and internal time step of 1800 seconds was chosen for numerical stability. Model output was stored daily. Turbulent components (4.9 m^2^ s^−1^) are related to the spatial resolution of the computational grid^64^. For all experiments, spawning regions are located in the northern part of SoS (see red polygons in Fig. 6). From them a spatially homogeneous (a regular grid of 2.6 by 3.3 km is used) set of 827 particles was released, with daily frequency.

In order to consider long-term hydrodynamic features and specific ages for larval recruitment the outcomes of the PTM were combined to obtain multiple settling windows and temporal ensembles scenarios of larval aggregation index (LA). LA indicates the fraction of the larvae that recruit over the total amount of larvae born annually and it was computed for each cell of the control grid as follows:

2 $$L{A}_{i}=\,\mathop{\sum }\limits_{j=1}^{n}{C}_{j}$$

where (*i)* indicates each cell of the recruitment areas, *C* the number of numerical particles entering a cell during the settling period and *(j)* the time range of the simulations. LA values, normalized by the maximum value obtained during the time range of the simulations, range between 0 and 1. The value *(n)* is the number of years included into each ensemble and can be modified to obtain seasonal, yearly or multiannual estimates.

The particles fluxes (PFs) were also computed with Eq. (3) and they were used to evaluate the origin of numerical particles that settle into defined recruitment regions. *C*_*R*_ is the number of numerical particles that settle in a recruitment region (*R*) during the time range of the simulations (*j)*. *C*_*R*_ is normalized by the number of particles (*N*_*S*_) that are released in the spawning region, during the whole simulation. As for LA, *(n)* can vary to obtain specific temporal ensemble of simulations.

3 $$PFs=\frac{1}{{N}_{S}\,\ast \,n}\mathop{\sum }\limits_{j=1}^{n}{C}_{Rj}$$

Two PTM experiments were set up to investigate the larval aggregation into known (i.e. derived from observations) and unexplored DPS nurseries as well as their connections with spawning regions (Table 1).

**Table 1:** Setup of the numerical simulations.

Name of the experiment	Name of the ensemble	Name list of model simulations
*I experiment*	*ENS 01*	*from 1994_1030 to 1997_1030*
		*from 1994_4060 to 1997_4060*
	*ENS 02*	*from 1998_1030 to 2010_1030*
		*from 1998_4060 to 2010_4060*
*II experiment*	*ENS 03*	*1995_1030*
		*1995_4060*
	*ENS 04*	*2004_1030*
		*2004_4060*

First column: the name of the experiment. Second column: the name of the computed ensemble. Third column: the name list of model simulations used to generate the ensemble scenarios (e.g. simulation 1994_1030 refers to the simulated year 1994 and the time window for settlement 10–30, the latter indicating the range of days after spawning that is considered valid for potential settlement of larvae).

The first experiment was aimed to reproduce the larval aggregation within the boundaries of known nursery areas located in the northern part of the SoS. Model results were compared to survey data and analysed to assess whether the connections between spawning and nursery areas were affected by the decadal variability of the SoS sea current system. Spawning (red polygons) and nursery areas (blue crossed lines), derived by the scientific trawl surveys dataset^54^ (Fig. 6), were used as input data for the PTM in order to define particles release areas and recruitment areas (i.e. where particle can potentially settle during the model simulation). The PTM run with 17 years of hydrodynamic hindcast (1994–2010) while numerical particles were released daily from the spawning areas on a regular grid (black dots in Fig. 6). Spawning and nursery areas were grouped into macro regions WEST, EAST and S-EAST (dotted lines in Fig. 6).

In the second experiment spawning areas were the same as for the first experiment. The boundaries of the recruitment areas were instead enlarged to include further DPS habitats that were identified on the base of the bathymetry and substrate preferences. Figure 4 defines them through coloured regions to simplify model outcomes representation and discussion. This second experiment was aimed to provide information on the long-distance connections that could occur between Siculo-Maltese spawning areas^26–28,55^, located in the northern sector of the SoS, and the entire domain of the SoS. The PTM run for two distinct years, 1995 and 2004, representing two states of the NIR that affects the SoS sea current system.

## Supplementary information

Supplementary material

## Data Availability

Connectivity matrices and geographical dataset of larval aggregation index will be made available at https://drive.google.com/open?id=1WZ8Di3R_Firf3XD7xE4MMzBcQe5ij1L5.
