# Supplementary material for: Hydrodynamic controls on connectivity of the high commercial value shrimp Parapenaeus longirostris (Lucas, 1846) in the Mediterranean Sea
Source: Sci Rep. 2019 Nov 15;9:16935. doi: 10.1038/s41598-019-53245-8 (PMC6858437; doi:10.1038/s41598-019-53245-8)
Supplement: Supplementary file 1 — Supplementary material [file 41598_2019_53245_MOESM1_ESM.docx]

**Hydrodynamic controls on connectivity of the high commercial value shrimp *Parapenaeus longirostris* (Lucas, 1846) in the Mediterranean Sea.**

Giovanni Quattrocchi^1^*, Matteo Sinerchia^1^, Francesco Colloca^2,3^, Fabio Fiorentino^2^, Germana Garofalo^2^ and Andrea Cucco^1^

^1^Institute for the study of Anthropic Impact and Sustainability in marine environment, National Research Council, Oristano, Italy.

^2^Institute for Marine Resources and Biotechnologies. National Research Council, Mazara del Vallo, Italy.

^3^Department of Biology and Biotechnology “C.Darwin”, Sapienza University of Rome, Italy.

Correspondence and requests for materials should be addressed to Giovanni Quattrocchi (email: giovanni.quattrocchi@ias.cnr.it)

**Supplementary material**

Supplementary material reports a sensitivity analysis that was carried out at the beginning of this research work set up. Before computing comprehensive LA index ensembles that were reported in the paper results, two ensemble scenarios were considered to understand how different pelagic larval duration of DPS could modify larval aggregation into the known nurseries of the northern part of the SoS.

The PTM simulations run during the period in between 1994 and 1997 and accounted for a fast and a slow larvae development scenario (i.e. at 10 and 40 days after release, numerical particles representing larvae have reached a suitable age for settlement).

The temporal ensemble (1994 – 1997; Fig. SM01) displays the recruitment into the known nurseries of the SoS as geographical distribution of the LA index when the time window for recruitment is in between 10 and 30 days after particle release.

In this case numerical particles can settle already at 10 days after release thus start aggregating in correspondence of the main nursery of the western side of the model domain. The strength of the Atlantic Ionian Stream flowing toward east and its latitudinal location allow for recruitment in correspondence of the nursery areas located offshore the southeast Sicilian coasts. LA values are high and very high in wide areas of the known nurseries.

The temporal ensemble (1994 – 1997; Fig. SM02) displays the recruitment into the known nurseries of the SoS as geographical distribution of the LA index when the time window for recruitment is in between 40 and 60 days after particle release.

In this case numerical particles can settle later, at 40 days, when the main sea current stream transported numerical particles far away from the western recruitment region of the northern part of the SoS. Very low values of LA resulted at west, while high to very high values indicate potential recruitment at the south and easternmost part of the nurseries that are located in correspondence of the Matlese Plateau.

| 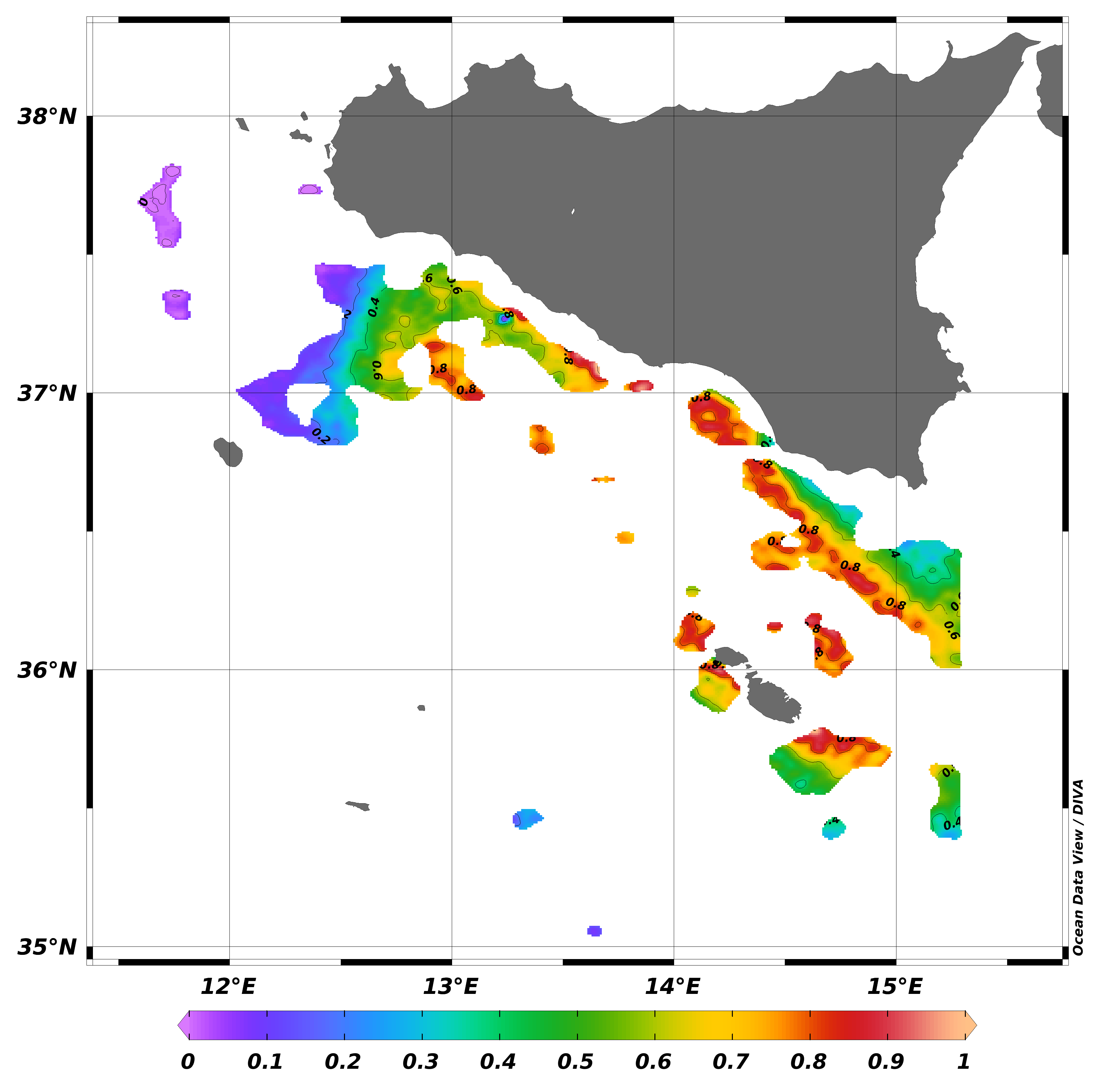 |
| --- |
| **Figure SM01** Larval Aggregation index map (LA) as generated by a first PTM sensitive experiment. The PTM run for the period 1994 and 1997 and for particle recruitment adopted a temporal window in between10 and 30 days after spawning. |

| 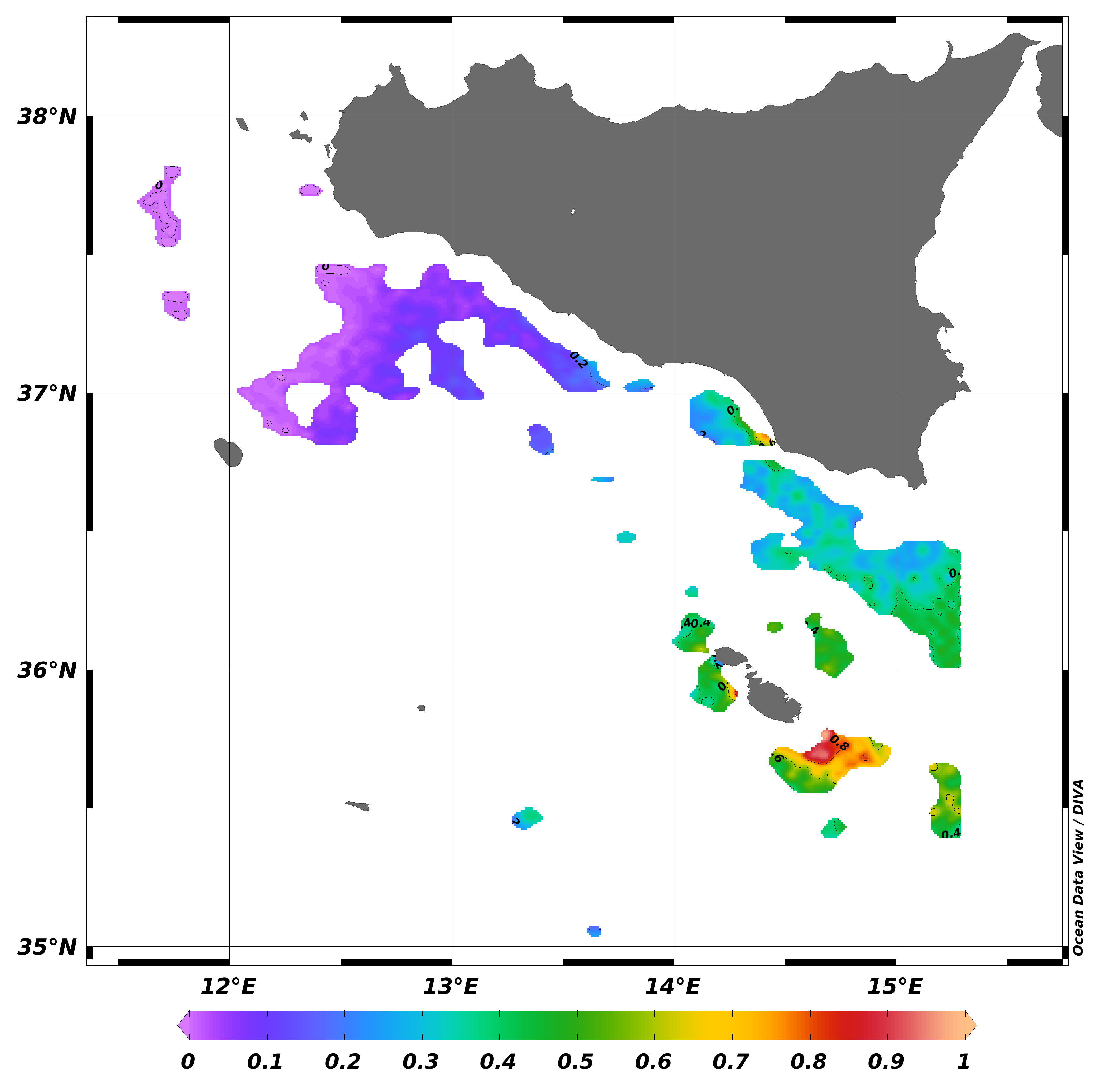 |
| --- |
| **Figure SM02** Larval Aggregation index map (LA) as generated by a first PTM sensitive experiment. The PTM run for the period 1994 and 1997 and for particle recruitment adopted a temporal window in between 40 and 60 days after spawning. |
